# Simultaneous Characterization of Wildfire Smoke and Surface Properties With Imaging Spectroscopy During the FIREX‐AQ Field Campaign

**DOI:** 10.1029/2021JD034905

**Published:** 2022-04-07

**Authors:** Philip G. Brodrick, David R. Thompson, Michael J. Garay, David M. Giles, Brent N. Holben, Olga V. Kalashnikova

**Affiliations:** ^1^ Jet Propulsion Laboratory California Institute of Technology Pasadena CA USA; ^2^ Science Systems and Applications Inc. (SSAI) Lanham MD USA; ^3^ NASA Goddard Space Flight Center (GSFC) Greenbelt MD USA

**Keywords:** imaging spectroscopy, AVCL, optimal estimation, surface reflectance

## Abstract

We introduce and evaluate an approach for the simultaneous retrieval of aerosol and surface properties from Airborne Visible/Infrared Imaging Spectrometer Classic (AVIRIS‐C) data collected during wildfires. The joint National Aeronautics and Space Administration (NASA) National Oceanic and Atmospheric Administration Fire Influence on Regional to Global Environments and Air Quality field campaign took place in August 2019, and involved two aircraft and coordinated ground‐based observations. The AVIRIS‐C instrument acquired data from onboard NASA's high altitude ER‐2 research aircraft, coincident in space and time with aerosol observations obtained from the Aerosol Robotic Network (AERONET) DRAGON mobile platform in the smoke plume downwind of the Williams Flats Fire in northern Washington in August 2019. Observations in this smoke plume were used to assess the capacity of optimal‐estimation based retrievals to simultaneously estimate aerosol optical depth (AOD) and surface reflectance from Visible Shortwave Infrared (VSWIR) imaging spectroscopy. Radiative transfer modeling of the sensitivities in spectral information collected over smoke reveal the potential capacity of high spectral resolution retrievals to distinguish between sulfate and smoke aerosol models, as well as sensitivity to the aerosol size distribution. Comparison with ground‐based AERONET observations demonstrates that AVIRIS‐C retrievals of AOD compare favorably with direct sun AOD measurements. Our analyses suggest that spectral information collected from the full VSWIR spectral interval, not just the shortest wavelengths, enables accurate retrievals. We use this approach to continuously map both aerosols and surface reflectance at high spatial resolution across heterogeneous terrain, even under relatively high AOD conditions associated with wildfire smoke.

## Key Points

- Imaging spectroscopy enables continuous maps of smoke aerosol and surface properties across heterogeneous terrain and dense plumes
- Information content analyses reveal sensitivity of imaging spectroscopy to broad aerosol categories
- Spectra from the complete Visible Shortwave Infrared range, including the shortwave infrared regions, contribute to accurate aerosol characterizations

## 1 Introduction

Atmospheric aerosols are fundamental to the physics and chemistry of the Earth's atmosphere and play important roles in the planetary radiation balance, the hydrologic cycle, atmospheric circulation, and even human health. Besides being one of the largest uncertainties in estimates of the future global climate (Boucher et al., 2013), the effects of aerosols in the present atmosphere are complex and often poorly understood (e.g., Kuniyal & Guleria, 2019). Climate change may also alter the relative concentrations and distributions of atmospheric aerosols through processes such as the desertification of potential dust sources (Green et al., 2020) and an increased incidence of wildfires (Barbero et al., 2015). New and improved measurements of aerosol quantity, size, shape, and chemical composition are necessary in order to monitor these sources and to better understand the processes of aerosol emission and transport. As aerosols vary widely in concentration and composition over space and time, observations from passive optical instruments with synoptic coverage from satellites will play a critical role in this effort.

A key challenge in measuring aerosols with passive remote sensing from a single‐angle view is the separation of atmospheric effects from the surface‐reflected radiance, especially over land. Spaceborne imaging sensors such as the Ozone Monitoring Instrument and the Moderate Resolution Imaging Spectroradiometer (MODIS) have exploited spectral observations in different wavelengths in the ultraviolet and visible (VIS) to shortwave infrared (SWIR), respectively, to retrieve aerosol optical depth (AOD), which is the total amount of aerosols in the atmospheric column, and some information about aerosol type, especially absorption (e.g., Buchard et al., 2015; Hsu et al., 2013; Levy et al., 2013; Sayer et al., 2014; Torres et al., 2007). Due to the complexity of the underlying surface, these algorithms often limit aerosol retrievals to wavelengths where the surface signal is expected to be low and, further, assume a simple statistical relationship—typically linear—between key wavelengths. Spatial averaging and preconditioning are also necessary to reduce the noise in the observations. These approaches are necessary because a handful of spectral channels are numerically insufficient to determine the surface/atmosphere separation. Unfortunately, the Earth's surface does not always adhere to such strict relationships, nor is it always possible to find nearby dark surface, which are among the challenges for these multiband approaches.

While the atmospheric science community is interested in aerosols for the reasons outlined above, the land surface community considers the presence of an overlying layer of aerosols a nuisance that must be removed in order to retrieve key information about surface ecology, biodiversity, mineralogy, vegetation health, and other geophysical parameters (e.g., Lee et al., 2015; Rast & Painter, 2019). This led to the development of “atmospheric correction” approaches, initially for multiband imagers. These techniques adapted for imaging spectrometers—also called hyperspectral imagers, due to their high spectral resolution and large number of spectral bands—to obtain accurate surface information with little attention paid to the details of the atmospheric aerosol (e.g., Gao et al., 2009; Rast & Painter, 2019; Thompson, Guanter, et al., 2019). However, recent work has leveraged the substantial information content of VIS to SWIR (VSWIR) imaging spectroscopy with high spectral resolution (≤10 nm) to simultaneously retrieve accurate surface and atmosphere states over heterogeneous terrain (Thompson et al., 2018, Thompson, Babu et al., 2019). A similar approach has demonstrated the capacity to retrieve atmospheric optical depths from extremely high spectral resolution (0.14/0.28 nm) data in the 290–695 nm region (Hou et al., 2016, 2017, 2020). In this study, we extend this approach to wildfire smoke with realistic constraints on physically possible surface reflectances and demonstrate the ability to accurately retrieve AODs from 0 to above 2 in the mid‐visible (550 nm) while showing sensitivity to aerosol optical properties at unprecedented spatial resolution.

The wildfire cases are taken from the western phase of the joint National Aeronautics and Space Administration (NASA) and National Oceanic and Atmospheric Administration (NOAA) Fire Influence on Regional to Global Environments and Air Quality (FIREX‐AQ) field campaign that took place in August 2019. A diverse suite of in situ and remote sensing instruments were deployed during this campaign. Here, we focus on data from NASA's “Classic” Airborne Remote Visible Infrared Imaging Spectrometer (AVIRIS‐C), which flew on the ER‐2 high altitude research aircraft, and coincident ground‐based sun photometer observations made by the Aerosol Robotic Network (AERONET). Simultaneous surface‐atmosphere retrievals using AVIRIS‐C data were performed using multiple aerosol models, demonstrating the ability to accurately retrieve AOD in comparison with AERONET and distinguish broad aerosol types using imaging spectroscopy in the VSWIR. These retrievals were performed at high resolution (16.3 m) to generate spatially continuous aerosol and atmospherically corrected surface maps. We further evaluate the information content of spectroscopic observations and show that aerosol related information is both dependent on the statistical constraints applied to the spectral surface reflectance, and distributed across the entire VSWIR spectral range. We close with a discussion of the implications of this work for imaging spectroscopy on NASA's upcoming Plankton, Aerosol, Cloud and ocean Ecosystem, Earth surface Mineral dust source InvesTigation (EMIT), Aerosol and Cloud, Convection and Precipitation (ACCP), and Surface Biology and Geology (SBG) satellite missions.

## 2 Methods

The joint NASA/NOAA FIREX‐AQ field campaign was designed to improve our understanding of the impacts of landscape fires (i.e., wildfires and controlled/agricultural burns) on climate, weather, and downwind air quality. During the western phase of the campaign in August 2019, the NASA high‐altitude ER‐2 research aircraft flew 11 flights over targets in Washington, Oregon, California, Utah, and Arizona from the NASA Armstrong Flight Research Center located in Palmdale, CA. Additional NASA and NOAA aircraft participated in the campaign, along with dedicated deployments of ground‐based stationary and mobile sensors. In this section, we describe the instruments and approaches used to retrieve and validate combined surface and atmospheric parameters from VSWIR imaging spectroscopy during FIREX‐AQ.

### 2.1 Airborne Measurements

During FIREX‐AQ, NASA's AVIRIS‐C flew in the Q‐bay located in the belly of the ER‐2 high‐altitude research aircraft. AVIRIS‐C measures radiance in 224 contiguous bands in the spectral range from 380 to 2,500 nm, with approximately 10 nm spectral sampling (Green et al., 1998). From the 20 km operational altitude of the ER‐2, the approximately one milliradian instantaneous field of view of AVIRIS‐C translates to 16.3 m ground‐level spatial sampling with a swath of about 11 km. The instrument is a whiskbroom imager with an oscillating scan mirror that sweeps across the 30° cross‐track field of view at 12 Hz, acquiring thousands of spectra per second. With this configuration, light from each cross‐track element passes through the same optical system, providing uniformity across the image swath. Four optical fibers route the light from the foreoptics into four spectrometers with the following spectral ranges (a) 380–700 nm (b) 700–1,300 nm (c) 1,300–1,900 nm, and (d) 1,900–2,500 nm. This approach allows each detector to be individually optimized (Green et al., 1998).

Prior to the campaign, AVIRIS‐C was laboratory calibrated using measurements of International System of Units traceable sources. During the campaign, the laboratory calibration was updated and refined using vicarious calibration from overflights of the Railroad Valley Playa, a dry lake bed in Nevada (Bruegge et al., 2021). A ground team made measurements of the surface of the playa on 4 August 2019, about 10 days prior to ER‐2 overflights on 13 and 15 August 2019. The shape of the reflectance of the playa is known to be stable within a few percent over multiple years, and vicarious calibration for Railroad Valley has an uncertainty of about 3% under ideal, clear sky conditions (Bruegge et al., 2019). Details of the vicarious calibration of AVIRIS‐C for FIREX‐AQ can be found in Bruegge et al. (2021). The resulting calibration coefficients were applied to the AVIRIS‐C data used in this investigation, rescaling the data to absolute radiance units. The resulting radiance cubes were geolocated using a camera model combined with on‐board GPS telemetry and mapped to a square, rectilinear grid with 16.3 m pixels. The same grid was used for aerosol retrievals and comparisons with ground‐based measurements.

### 2.2 Ground‐Based Measurements

The AERONET is a distributed network of ground‐based sun photometers that provide information about atmospheric aerosol loading (AOD) and aerosol properties by measuring direct solar intensity and directional sky radiances in a number of visible and near‐infrared wavelengths (Dubovik & King, 2000; Giles et al., 2019; Holben et al., 1998; Sinyuk et al., 2020). In addition to the static AERONET sites, during FIREX‐AQ specially modified sun photometers were mounted on two vehicles and attempts were made to place these vehicles under wildfire smoke plumes to measure their aerosol properties and serve as validation for remote sensing retrievals (Holben et al., 2018). This was accomplished successfully for the Williams Flats Fire that burned on the Colville Indian Reservation, about 80 km northwest of Spokane, WA (e.g., Junghenn Noyes et al., 2020).

Table 1 lists the coincident measurements between AVIRIS‐C and AERONET identified during the FIREX‐AQ campaign. We gathered all instance of data where acquisitions were less than 100 m apart (AVIRIS pixel center compared to AERONET location), and also less than 15 min apart. In all cases, the closest match to AVIRIS‐C was within a single retrieval pixel (≤16.3 m), and the dates and times reported are the closest matching AERONET instance. AERONET AODs were linearly interpolated in log‐log space to 550 nm using the two nearest AERONET wavelengths on either side of the desired wavelength (e.g., Sayer et al., 2013). Note that not all the matches were for conditions with wildfire smoke.

**Table 1:** Airborne Visible/Infrared Imaging Spectrometer Classic Collocations With Aerosol Robotic Network (AERONET) Sites During Fire Influence on Regional to Global Environments and Air Quality in 2019

AERONET Site	Date	AERONET Min Time (UTC)	AERONET Closest Time (UTC)	AERONET Max Time (UTC)	AVIRIS Time (UTC)	Lat (°N)	Lon (°W)
Mobile 2	08/06	18:27:03	18:41:55	18:47:17	18:41:54	47.9110	118.3350
Mobile 2	08/06	20:24:34	20:38:55	20:54:18	20:39:22	48.1020	118.2060
Mobile 2	08/06	21:00:52	21:12:29	21:12:29	21:15:49	48.1020	118.2060
Mobile 1	08/07	18:14:50	18:27:52	18:29:50	18:28:43	47.9061	118.3337
CalTech	08/12	18:51:58	19:06:58	19:18:58	19:05:38	34.1367	118.1262
UFR	08/21	22:51:51	23:03:44	23:12:44	23:04:10	35.2148	111.6344
UFR	08/21	23:06:43	23:06:43	23:33:43	23:19:07	35.2148	111.6344

*Note.* *UFR stands for USGS Flagstaff ROLO.

### 2.3 Retrieval Strategy

Surface and atmospheric properties were simultaneously estimated using a Bayesian Maximum A Posteriori (MAP) inversion approach. In the satellite remote sensing and atmospheric science communities, this is known colloquially as Optimal Estimation (OE; e.g., Maahn et al., 2020; Nguyen et al., 2019; Rodgers, 2000). Recently, the method was adapted for retrievals using imaging spectroscopy data from the AVIRIS‐Next Generation (AVIRIS‐NG) instrument (Thompson et al., 2018, Thompson, Babu et al., 2019). In comparison to AVIRIS‐C, AVIRIS‐NG has nearly twice as many spectral samples (425 versus 224) within the spectral range from 380 to 2,510 nm (Chapman et al., 2019). One of the goals of the present work is to demonstrate the OE approach using the lower spectral resolution data from AVIRIS‐C. In this section we summarize the salient points regarding the application of OE to AVIRIS‐C aerosol retrievals for FIREX‐AQ cases. More in‐depth technical discussions of OE retrievals for imaging spectroscopy can be found in Thompson et al. (2018); Thompson, Babu et al. (2019).

We begin with a *state vector*, x, that represents the set of surface, **x**_**s**_, and atmospheric, **x**_**a**_, parameters we wish to estimate using the AVIRIS‐C observations. In the specific cases considered here, **x**_**s**_ represents the Lambertian surface reflectances for all 224 AVIRIS‐C spectral bands. The atmospheric state, **x**_**a**_, includes AOD at 550 nm of one or more aerosol types and the column water vapor concentration. For convenience, we further represent the known solar and sensor geometry as an additional vector, **g**. A *forward model*, **f**, maps the state vector to an estimate of the radiance at the sensor, ${\hat{l}}_{o}=f(x,g)+\epsilon$, where ***ϵ*** is a vector of measurement errors that are assumed Gaussian and independent of the state vector, **x**.

Making the simplifying assumption of a locally homogeneous, Lambertian surface (e.g., Tanré et al., 1979; Lee & Kaufman, 1986; Pinty et al., 2005), the forward model can be written as:

(1) $${\hat{l}}_{o}=l_{\mathrm{atm}}\left(x_{a},g\right)+\left[l_{\text{dn}}(g)\cdot \tau \left(x_{a},g\right)\cdot r\left(x_{s}\right)\right]\cdot \frac{1}{1-s\left(x_{a},g\right)\cdot r\left(x_{s}\right)}+\epsilon .$$

The first term, **l**_**atm**_, is the *atmospheric path radiance*, which represents light scattered by the atmosphere back into the sensor that never interacts with the surface, and carries most of the information about the aerosol and water vapor content of the atmospheric column. The term in brackets contains the total (direct + diffuse) downwelling irradiance at the surface, **l**_**dn**_, that is attenuated by transmission through the atmosphere, ***τ***, and reflected by a single bounce from the surface, which has a hemispherical‐directional reflectance factor (HDRF), given by **r**. The HDRF is the ratio of the reflected radiant flux from the surface due to the incoming light from the entire hemisphere to the reflected radiant flux from an ideal, diffusely reflecting (Lambertian) surface (Schaepman‐Strub et al., 2006). If the surface was such a perfectly diffusely reflecting surface, then **r** ≡ 1. While, in practice, the HDRF of the surface is much less than one, the effect on the modeled reflectance for the surface modeled here is effectively that of a scaler, which will not impede the AOD retrievals. For simplicity, we will henceforth refer to the HDRF as the surface reflectance or just reflectance. The set of surface reflectances for the AVIRIS‐C wavelengths corresponds exactly to the surface state vector, **x**_**s**_. The fraction that appears after the brackets accounts for multiple scattering, which is light that interacts with the surface and the atmosphere multiple times. Each interaction modifies the term in the brackets by a multiple of the spherical albedo of the atmosphere observed from the ground, **s**, and the light diffusely reflected upward from the surface, **r**. The sum of these interactions make up a geometric series that is represented by the fraction in the limit of an infinite number of interactions. Finally, the measurement noise, ***ϵ***, is assumed to be Gaussian with a zero mean and a covariance given by **Σ**_*e*_. Note that additional terms could be included to account for surface emission, which may be important for very hot targets, like active fires. However, since direct measurements of the hot fire front were very sparse, these terms were not used.

The OE retrieval approach uses Bayes' theorem to estimate the state vector, including both surface and atmosphere terms, most likely to have yielded the true observation **l**_**o**_, after taking into account both measurement noise and the strength of any prior information. Bayes' theorem is given by the expression:

(2) $$p\left(x|y\right)=\frac{p\left(y|x\right)p\left(x\right)}{p\left(y\right)}.$$

This equation should be read: the probability of a state, **x**, given by the observations, **y**, is equal to the probability of **y** given **x** times the probability of **x** divided by the probability of **y**. In words, Bayes' theorem states that the posterior probability, *p*(**x|y**), is equal to the likelihood, *p*(**y|x**), times the prior, *p*(**x**), divided by the evidence, *p*(**y**). The evidence, or the marginal likelihood, does not provide any information on the state vector **x**, so for practical purposes Bayes' theorem is simplified to:

(3) p(x|y)∝p(y|x)p(x).

in general, we take the prior to be a multivariate Gaussian distribution given by:

(4) $$p\left(x\right)\propto \exp\left[-\frac{1}{2}{\left(x-{\bar{x}}^{p}\right)}^{T}\Sigma_{p}^{-1}\left(x-{\bar{x}}^{p}\right)\right],$$

where ${\bar{x}}^{p}$ is the mean of the assumed prior distribution of the state vector with a covariance **Σ**_*p*_, and the superscript *T* designates the transpose of the vector. Note that the term in the brackets is the square of the Mahalanobis distance, which is a multidimensional generalization of the Euclidian distance (De Maesschalck et al., 2000). In a similar fashion, the difference between the modeled and sensor observations, sometimes called the “noise,” but which actually contains both the error in the forward model and the measurement noise, is expressed in Gaussian form as:

(5) $$p(y|x)\propto \exp\left[-\frac{1}{2}{\left(l_{o}-{\hat{l}}_{o}\right)}^{T}\Sigma_{e}^{-1}\left(l_{o}-{\hat{l}}_{o}\right)\right],$$

where l_**o**_ is the true observation, ${\hat{l}}_{o}$ is the modeled observation from the forward model, and **Σ**_*e*_ is the error covariance matrix. With these assumptions, the posterior probability becomes:

(6) $$p\left(x|y\right)\propto \exp\left[-\frac{1}{2}{\left(l_{o}-{\hat{l}}_{o}\right)}^{T}\Sigma_{e}^{-1}\left(l_{o}-{\hat{l}}_{o}\right)\right]\left[-\frac{1}{2}{\left(x-{\bar{x}}^{p}\right)}^{T}\Sigma_{p}^{-1}\left(x-{\bar{x}}^{p}\right)\right].$$

Taking the logarithm of both sides, we obtain:

(7) $$\chi^{2}(x)\equiv -2\ln p(x|y)=\left[{\left(l_{o}-{\hat{l}}_{o}\right)}^{T}\Sigma_{e}^{-1}\left(l_{o}-{\hat{l}}_{o}\right)\right]+\left[{\left(x-{\bar{x}}^{p}\right)}^{T}\Sigma_{p}^{-1}\left(x-{\bar{x}}^{p}\right)\right],$$

which is the OE cost function (Cressie, 2018). Minimizing this cost function leads to the MAP estimate, the most probable state that includes all the prior information and posterior probabilities (Thompson, Babu, et al., 2019).

In our implementation, the solution to Equation 7 is found using a trust‐region method, a common nonlinear gradient‐best optimization technique that guarantees local convergence for continuous problems (Branch et al., 1999; Conn et al., 2000). Columns of the Jacobian corresponding to atmospheric state vector terms (water vapor and AOD) were estimated using finite differences of the look up table (see Section 2.4), while columns related to the surface were calculated analytically using the chain rule on Equation 1. Starting points were initialized near the atmospheric state bounds for water vapor and AOD for each aerosol type and the corresponding heuristically determined surface reflectance starting points, in order to help ensure a more global optimization. We found that the retrieval proved to be generally robust, with the multipoint initialization leading to spatially smooth atmospheric state values, consistent with expectation. Both the averaging kernel matrix—a representation of the sensitivity of the cost function to the true state—and an estimate of the uncertainty based on the full posterior predicted distribution, can be calculated at the retrieved state. Full descriptions of these calculations are derived in (Rodgers, 2000), and the exact formulation used here is available in (Thompson et al., 2018).

**Figure 1:**
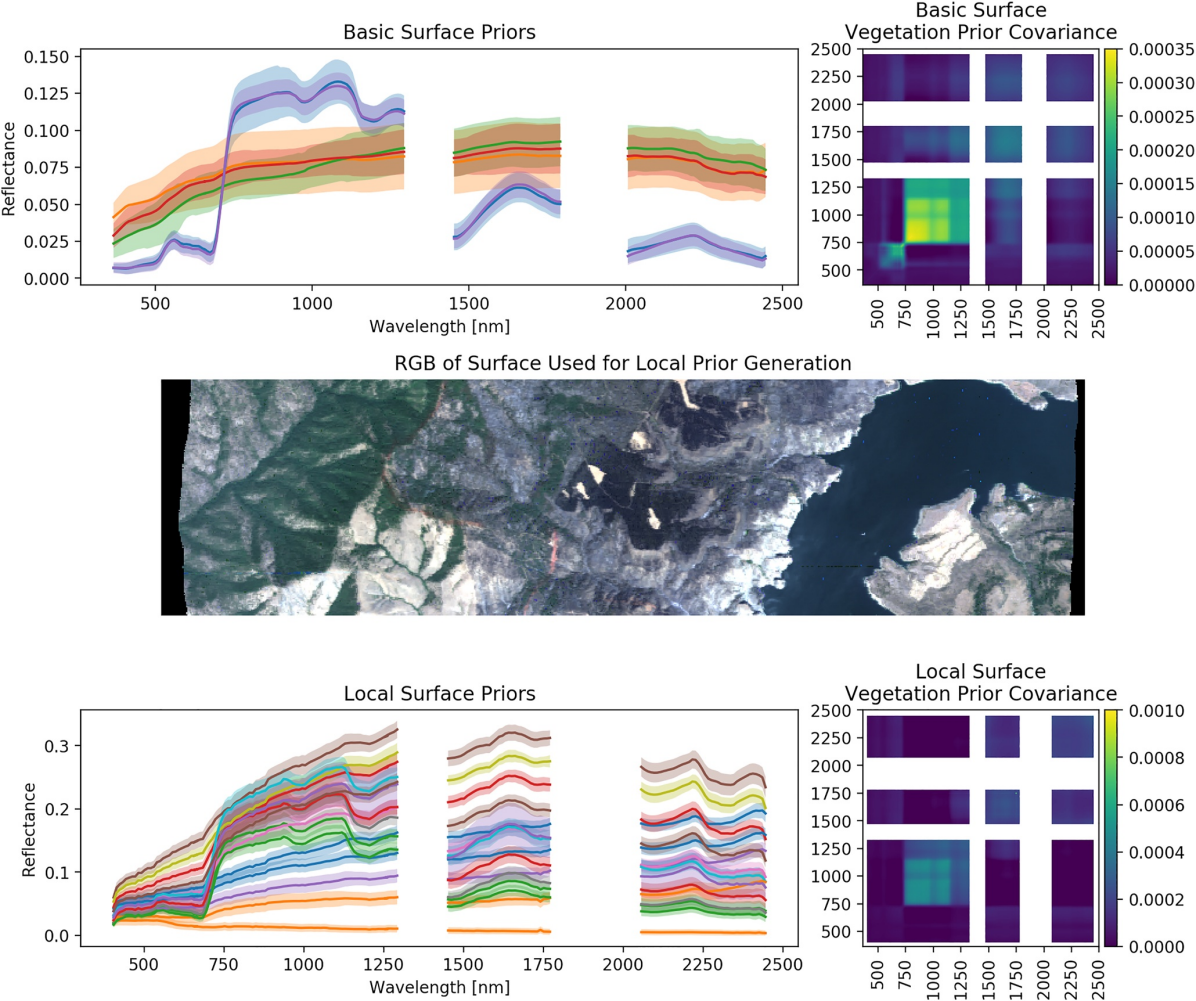
Illustration of basic (top) and localized (bottom) priors for the surface component of the state vector, **x**_**s**_. The priors shown in the top set are drawn from a moderately diverse set of reference spectra, as per Thompson et al. (2018). These were used to estimate the surface reflectance of a clear‐sky area of land (middle panel) located upwind of the target area of interest that contained the smoke plume. The resulting surface reflectances were then clustered into the local surface priors shown in the bottom panel. Each panel of priors shows the prior means as different colored lines and root mean square of the covariance on the left, and the full covariance matrix of a selected pixel on the right. White regions in the plots indicate spectral ranges that are dominated by water vapor and contain little information about the surface.

Returning to Equation 7, careful consideration reveals that the second term in square brackets, which includes the prior distribution, acts as a regularization parameter for the solution of an ill‐posed problem (Cressie, 2018; Nguyen et al., 2019). For our application, we exploit this characteristic of the prior in a two‐step manner to improve the performance of the algorithm under conditions of high aerosol loading where the underlying surface is partially or completely obscured at shorter wavelengths by the atmosphere. Recall that the surface model prior is based on a collection of multivariate Gaussian distributions, as shown in Equation 4. It is common in operational settings to use “universal” models that provide only very weak, or “soft,” constraints (Thompson et al., 2020). As illustrated in Figure 1, we performed an initial atmospheric correction using soft constraints from what we consider “universal surface models.” These are represented by the basic surface priors shown at the top of the figure, which have smoothly varying reflectances as a function of wavelength, with a broad spread about the mean, and very small band‐to‐band covariances peaking around 3.5 × 10^−4^. We then selected large, rectangular areas of heterogeneous terrain upwind of the smoke plumes, where the retrieval of the surface reflectance could be considered trustworthy. The surface reflectances were grouped using K‐means clustering, and we obtained a set of within‐group means and covariance matrices. These locally derived surface priors, associated spreads, and band‐to‐band covariances are shown in the bottom portion of Figure 1. Compared to the basic surface priors, the local surface priors have more mixture representation with much tighter agreement about the mean, and larger covariances, which ranges up to 1.0 × 10^−3^ for the selected pixel shown. We note that the magnitudes of the reflectance values (which differ between the two prior sets due to the different data sources used for each) are not important, as they are scaled uniformly during the retrieval. These stronger priors were then used in a second pass of the OE retrieval for the portion of the image obscured by the dense smoke plume in the shorter wavelengths.

### 2.4 Atmospheric Radiative Transfer

The complete forward model **f**(**x**, **g**) includes models of the sensor, surface, and atmosphere that transform state variables to a predicted radiance. The surface model is described in Section 2.3, and the instrument model contains a component‐wise description of the AVIRIS‐C sensor with constant noise terms that account for electronic and detector thermal effects, as well as signal‐dependent noise from photon counting statistics (Thompson et al., 2018). In this section, we describe the atmospheric models used.

In order to determine the optical coefficients used in Equation 1, we ran a series of MODTRAN 6.0.2.2 G radiative transfer model simulations for each scene (Berk & Hawes, 2017). While in theory the formulation in Section 2.3 can estimate any combination of atmospheric state parameters, in this work we focus on two key atmospheric components: the total column water vapor and the AOD for three different aerosol types. The three aerosol types used in this investigation were the sulfate and dust models previously used for AVIRIS‐NG aerosol retrievals over India (Thompson, Babu, et al., 2019) as well as a fine smoke aerosol model based on AERONET climatological observations (Omar et al., 2005, 2009). The sulfate model is based on Chin et al. (2002) and Hess et al. (1998). The dust model is taken from a single size bin from 1 to 1.8 *μ*m in the OPAC‐Spheroids model described in Colarco et al. (2014). The dust spectral refractive indices are based on the OPAC data (Hess et al., 1998), and the shape information is drawn from the nonspherical single scattering aerosol database described by Meng et al. (2010). The dust and sulfate models were not intended to represent particular species, but to encapsulate general optical properties of different classes (Thompson, Babu, et al., 2019).

The smoke model has a log‐normal size distribution given by:

(8) $$\frac{dn\left(r\right)}{d\;\ln\;r}=\frac{N_{0}}{\sqrt{2\pi }\cdot \ln\;\sigma }\cdot \exp\left[\frac{-{\left(\ln\;r-\ln\;r_{c}\right)}^{2}}{2{\left(\ln\;\sigma \right)}^{2}}\right],$$

where the left hand side of the equation describes the number of particles in equal steps in the logarithm of the radius, *r*, and *N*_0_ is a normalization term. The key parameters of the distribution are *r*_*c*_, the characteristic radius (sometimes called the modal radius), and *σ*, which is the characteristic width (sometimes call the geometric standard deviation). From Omar et al. (2005); Omar et al. (2009), *r*_*c*_ = 0.0790 *μ*m, and *σ* = 1.5624 *μ*m. Note that the characteristic radius is derived from the volume‐weighted characteristic radius, *r*_*v*_ distribution given for the fine mode smoke in Omar et al. (2005); Omar et al. (2009), using the conversion: *r*_*c*_ = *r*_*v*_ exp[−3 (ln *σ*)^2^] (Remer & Kaufman, 1998).

Omar et al. (2009) provide the real and imaginary part of the index of refraction at two wavelengths, 532 and 1,064 nm, since the model is derived for use with the Cloud‐Aerosol Lidar and Infrared Pathfinder Satellite Observations (CALIPSO) aerosol products. These values *n*_*r*_(532) = 1.517, *n*_*r*_(1,064) = 1.541, for the real part, and *n*_*i*_(532) = 0.0234, *n*_*i*_(1,064) = 0.0298 were interpolated in log‐log space to the required MODTRAN wavelengths. The difference between a simple linear interpolation and the log‐log interpolation is small for the AVIRIS‐C wavelengths used in the retrievals. Single scattering properties were calculated using a Mie code assuming spherical particles (Mishchenko et al., 1999).

**Figure 2:**
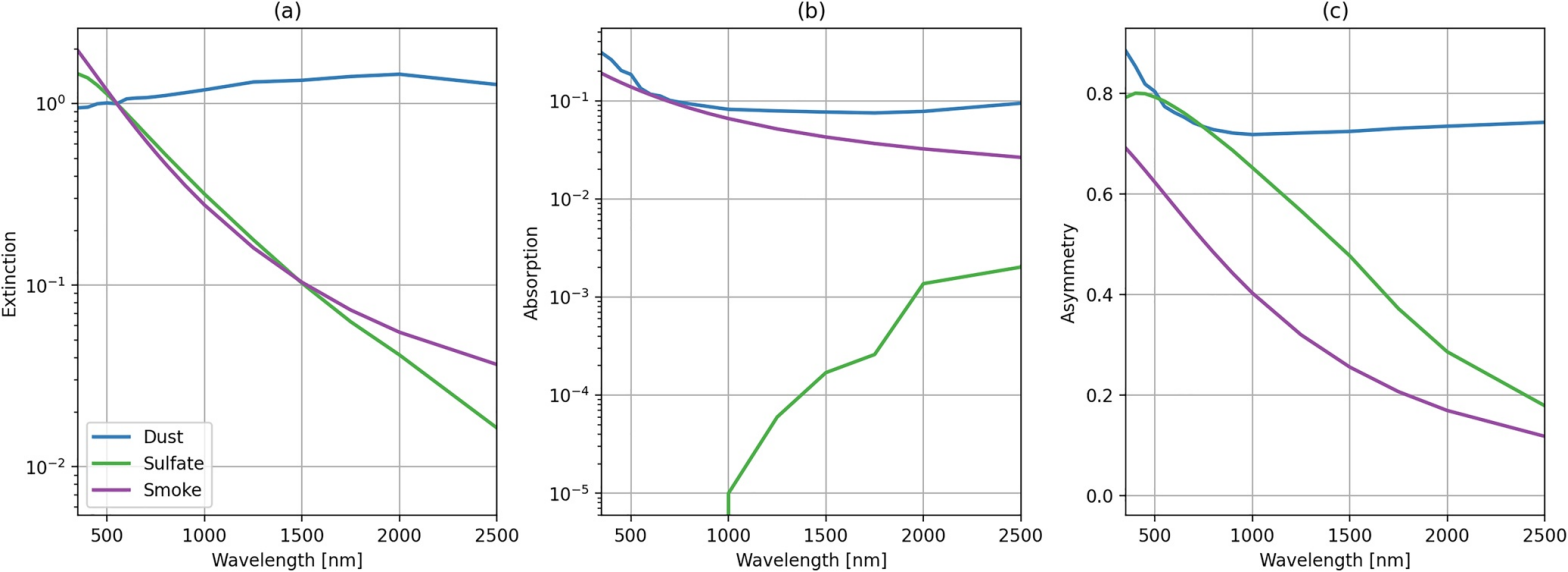
Aerosol model components for different aerosol types as a function of wavelength, showing (a) the normalized extinction coefficients, (b) the absorption coefficients, and (c) the asymmetry parameters for the three aerosol models. Dust is indicated in blue, sulfate in green, and smoke in purple.

The extinction, absorption, and asymmetry parameters of each aerosol are shown as a function of wavelength in Figure 2. These are the key parameters used in the atmospheric radiative transfer performed by MODTRAN (Berk & Hawes, 2017). This figure demonstrates that the sulfate and smoke scattering coefficients are very similar due to similar size distributions. Their absorption coefficients, however, differ significantly in the 0.4–2.5 *μ*m range. By comparison, the dust spectral optical properties differ significantly from those of the other two aerosol models. Although the dust model is used in the simulation experiment described in the next section, detailed investigation of AVIRIS‐C sensitivity to atmospheric dust is beyond the scope of this investigation, which is focused on fire observations.

Given the aerosol properties, MODTRAN 6.0 was then used to calculate the optical properties ***τ***, **s**, and **l**_**atm**_ that appear in Equation 1 using the mean view and solar angle geometries for each scene. As in Thompson et al. (2018), the simulations were run using the correlated‐k representation to handle atmospheric absorption with 17 coefficients per 0.1 cm^−1^ spectral bin. Vertical distributions of constituents assigned according to the MODTRAN midlatitude summer profile. Multiple scattering was performed using the DISORT (Stamnes et al., 1988) method internal to MODTRAN, with 8 streams (Berk & Hawes, 2017). We note that MODTRAN does not account for polarization effects, which may play a significant role, particularly below 500 nm. The resulting coefficients were placed in a lookup table (LUT) indexed by atmospheric state. AOD values in the LUT for each aerosol type ranged from 0 to 3 with six evenly spaced values. Interpolations within the LUT were used to determine the precise radiance for any given state vector during individual pixel inversions (Thompson, Babu, et al., 2019).

## 3 Results

We first present a small series of simulation results to provide intuition about the effects of different aerosols on at‐sensor radiance for AVIRIS‐C, followed by retrievals of AODs over multiple locations from the FIREX‐AQ campaign and comparisons with AERONET.

### 3.1 Simulation Comparisons

**Figure 3:**
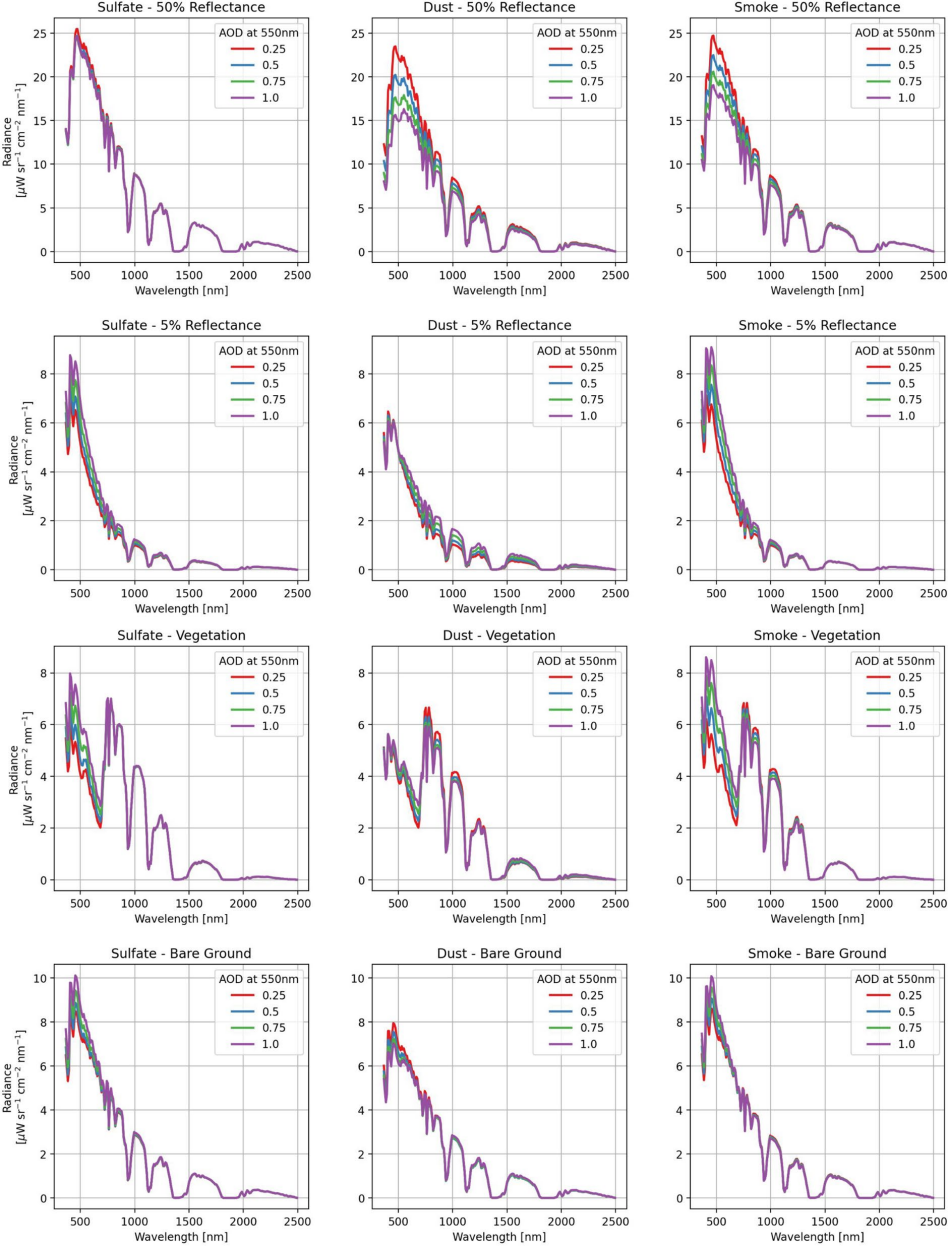
Simulated at‐sensor radiance for different targets (top row: uniform 50%, second row: uniform 5%, third row: vegetation, and fourth row: bare ground), for the three different aerosol types (columns). Each of the four different aerosol optical depths ranging from 0.25 to 1.0 at 550 nm are shown in each panel. Note that the rows use a constant scale for the *y*‐axis, but the scales are different from the top row to the bottom.

We begin by showing the absolute at‐sensor radiances, modeled using Equation 1, for an arbitrary bright and dark target (uniform reflectances of either 50% or 5%), as well as for a vegetation and a bare ground spectrum. Keeping the amount of atmospheric water vapor fixed to 2 g cm^−2^, we varied AOD values for each aerosol independently from 0.25 to 1.0. The results are shown in Figure 3. For the bright surface in the top row, the absorbing aerosols (dust and smoke) dramatically affect the at‐sensor radiances, especially around 500 nm. Larger effects are seen with higher AOD. This sensitivity to absorbing aerosols over bright surfaces is the basis for the “critical reflectance” approach for retrieving aerosol single scattering albedo (e.g., Seidel & Popp, 2012; Wells et al., 2012; Zhu et al., 2011). The situation is different for the dark surface, where the smoke aerosol has the largest at‐sensor radiances around 500 nm. To first order, this is due to the smaller asymmetry parameter for the smoke aerosol model as shown in Figure 2, which indicates less scattering in the forward direction and, consequently, more backscattered light from the aerosol. It is also worth noting that the dust model shows the effects of changing AOD throughout the VSWIR spectral range. This is because the extinction coefficient is relatively constant for dust as a function of wavelength (Figure 2), due to the relatively large particle size of the dust model compared to the sulfate and smoke models. Nonuniform surface targets, such as vegetation or bare soil (bottom two rows of Figure 3), further complicated the modeled at‐sensor radiance features, though the distinction between aerosol models is still quite clear.

**Figure 4:**
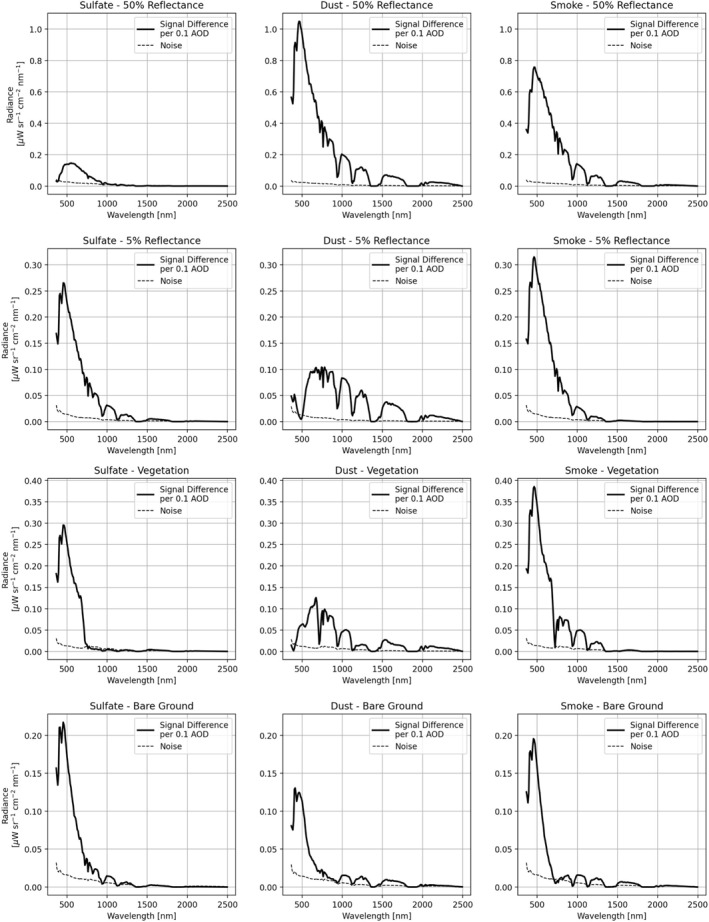
The mean change in at‐sensor radiance in the 0.25–1.0 aerosol optical depth (AOD) range, per 0.1 unit difference of AOD, relative to the simulated Airborne Visible/Infrared Imaging Spectrometer measurement noise. Simulations were performed using a uniform reflectance target of either 50% (top) or 5% (second row), along with an arbitrary vegetation spectrum (third row) and bare earth spectrum (fourth row) for different aerosol types. In all cases, an atmospheric water vapor value of 2.0 g cm^−2^ was used.

To further investigate the behavior of the at‐sensor radiances for different aerosol types, we used the same set up to calculate the mean radiance deviation per 0.1 unit change in AOD within the 0.25 to 1.0 AOD range can compared this to the estimated AVIRIS‐C noise (Figure 4). The different panels in this figure are often referred to as radiative kernels. This comparison highlights that the available signal from a 0.1 change in AOD typically exceeds the sensor noise threshold‐indicating that there is sufficient signal to make a detection. These results do not, however, determine whether or not a retrieval strategy will be able to distinguish between surface, AOD, and water vapor‐for that analysis we examine remote detections in the next section.

### 3.2 Remote Retrievals

**Figure 5:**
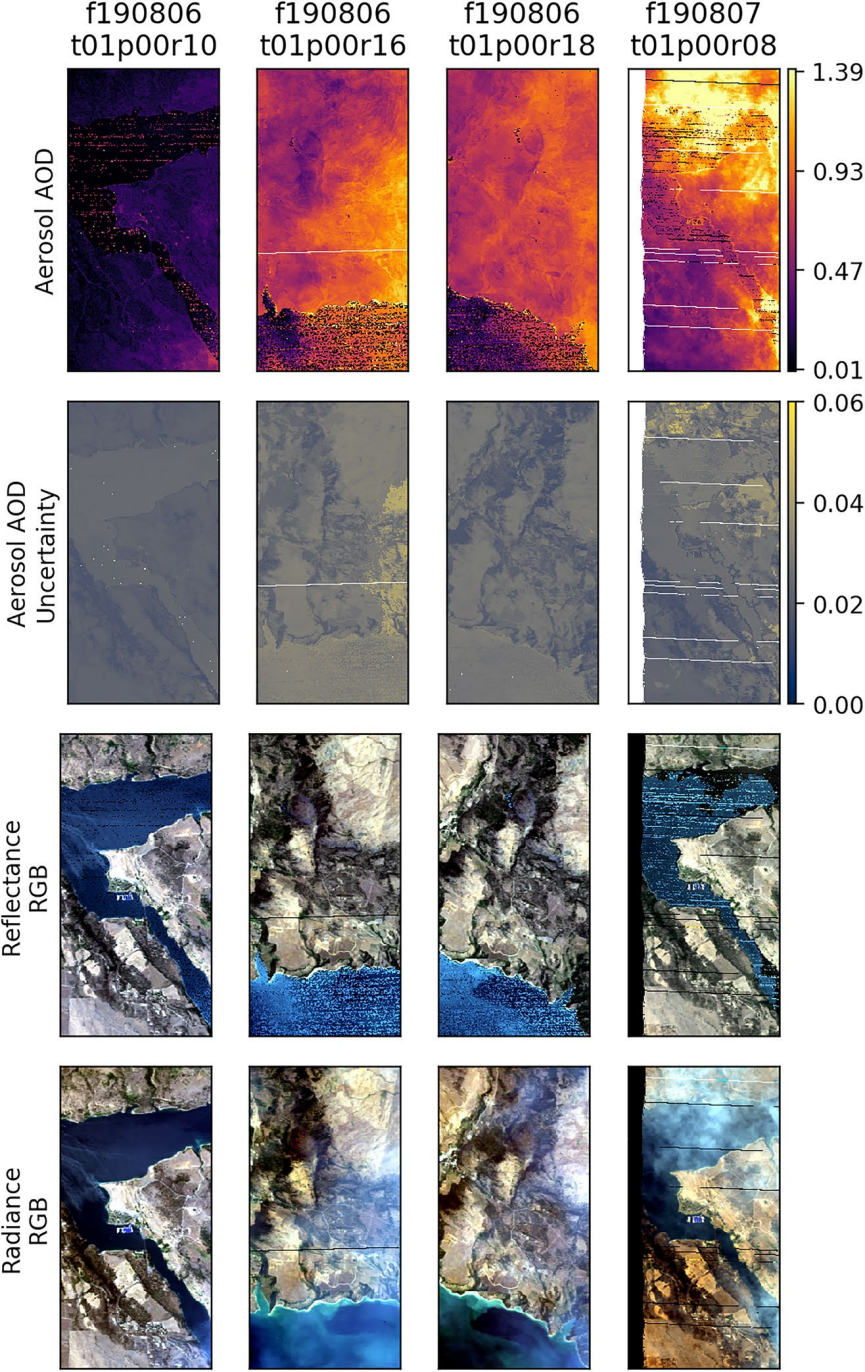
Mapped retrieval results over the mobile Aerosol Robotic Network (AERONET) locations from August 6 and 7, 2019. From top to bottom, figures show the aerosol aerosol optical depth (AOD) modeled by Optimal Estimation (using the Cloud‐Aerosol Lidar and Infrared Pathfinder Satellite Observations smoke model), the corresponding AOD uncertainty, an RGB image from the retrieved reflectance, and the initial radiance. The same area is visible in several scenes, observed at different points in time with different aerosol values. Each scene is a 200 × 400 pixel (3,200 × 6,400 m) area, centered on the mobile AERONET site.

We implemented the OE retrieval strategy described in Section 2.3 on all AVIRIS‐C acquisitions with spatially and temporally coincident AERONET mobile acquisitions. An example of these retrievals using the smoke model is shown in Figure 5. The top row shows the retrieved AODs for all four scenes, ranging from very low to very high amounts of aerosols. The second row shows the estimated AOD uncertainty (in units of AOD), which remains small relative to the aerosol levels present in these scenes. Careful inspection of the scenes indicates that the AOD uncertainties are lowest over more vegetated pixels and highest over the pixels with more bare ground, consistent with previous findings (Thompson, Babu, et al., 2019). The third row shows “atmospherically corrected” RGB images from the retrieved reflectances. For comparison, the last row provides RGB images of the measured at‐sensor radiance. It is apparent that the retrieval does a good job removing the presence of smoke, indicating a robust AOD retrieval using this aerosol model. Some retrieval instabilities are noticeable over water pixels where the observed radiances tend to be extremely low.

**Figure 6:**
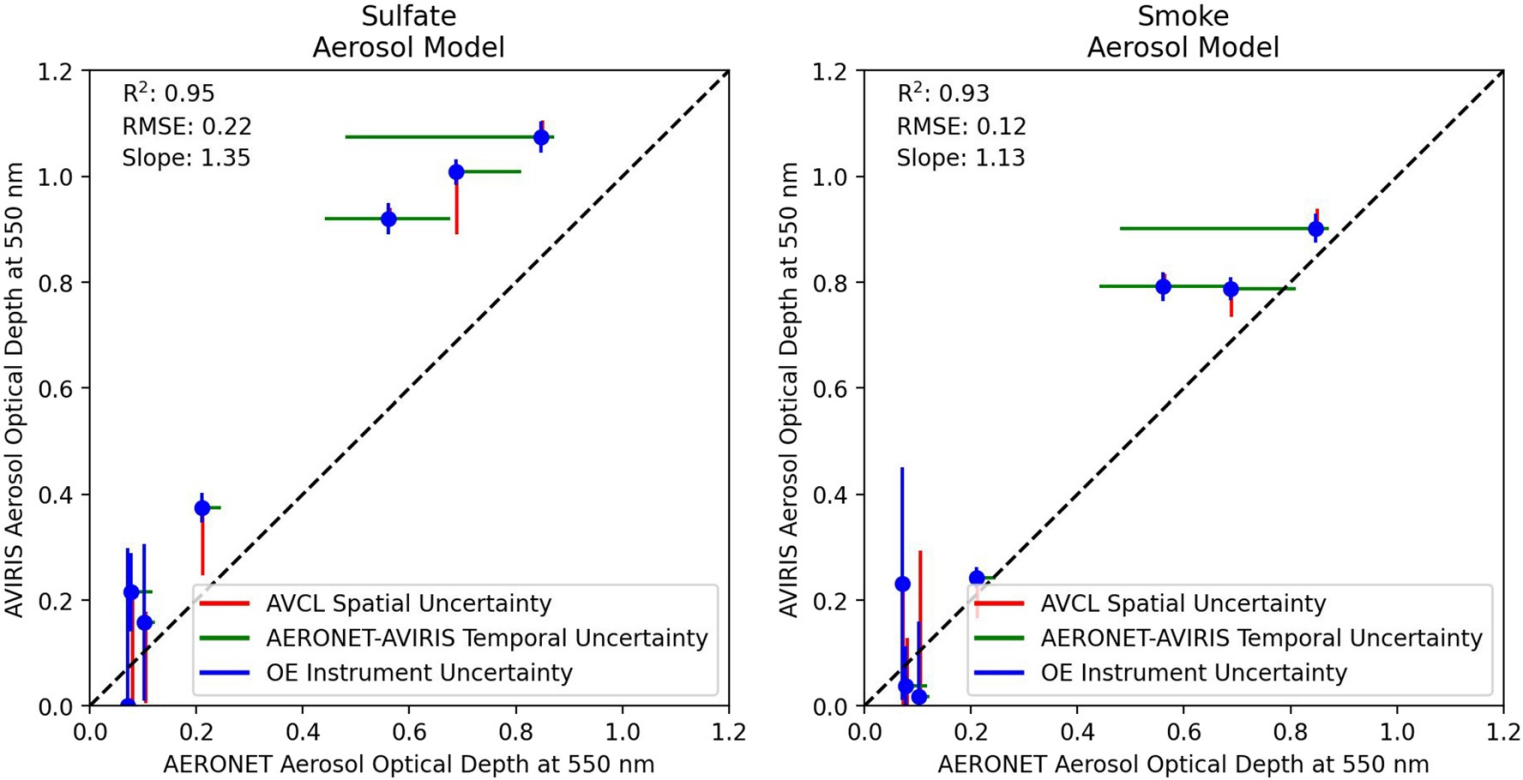
Comparison between aerosol optical depth (AOD) at 550 nm estimated through Optimal Estimation (OE) from the Airborne Visible/Infrared Imaging Spectrometer Classic data, and AOD at 550 nm estimated from mobile Aerosol Robotic Network (AERONET) units. The range of values in the Airborne Visible/Infrared Imaging Spectrometer (AVIRIS) scene in the 3 × 3 pixel grid surrounding the target are shown as the spatial uncertainties, all AERONET values within the nearest 15 min of the time of acquisition of the target pixel are shown as AERONET‐AVIRIS temporal uncertainties, and the uncertainty from the optimal estimation AOD retrieval is shown as the OE instrument uncertainties. AERONET‐AVIRIS spatial and temporal uncertainties indicate potential uncertainty in the alignment between the two measurements. AERONET direct measurement uncertainty for the Version 3 Level 2.0 AOD measurements for mid‐visible wavelengths is very low, typically less than 0.01 (Eck et al., 1999; Giles et al., 2019), and so not shown directly.

In addition to retrievals over the mobile AERONET platform, we also ran similar retrievals over several fixed AERONET sites under clear‐sky conditions (see Table 1). Figure 6 shows a comparison of retrievals performed using MODTRAN radiative transfer simulations using both the sulfate and smoke aerosol models. The dust aerosol model unsurprisingly resulted in near‐zero AOD estimates, and is consequently excluded from subsequent analyses. AODs retrieved using both the smoke and sulfate aerosol models compare favorably with the limited number of spatially and temporally coincident data acquisitions from AERONET and AVIRIS‐C (Table 1). This is particularly true given the number of conflicting factors between measurements, which include viewing geometry differences as well as potential spatial and temporal misalignment. To help assess these, we display multiple metrics of uncertainty for each point. As each line was manually assessed for orthorectification errors, we expect the spatial alignment to be strong relative to the 16 m ground level resolution data. As such, we take the spatial uncertainty range to be the 3 × 3 pixel grid overlaying the target location, and plot the minimum and maximum values. While we expect the temporal accuracy of both instruments to be high, small timing offsets could result in relatively large changes in smoke plume location, and as such we show the 15 min interval around the closest matching mobile AERONET measurement. The center point, however, is the closest temporal match (corresponding to Table 1). Comparing the performance, the smoke model appears to show less bias relative to AERONET than the sulfate model.

**Figure 7:**
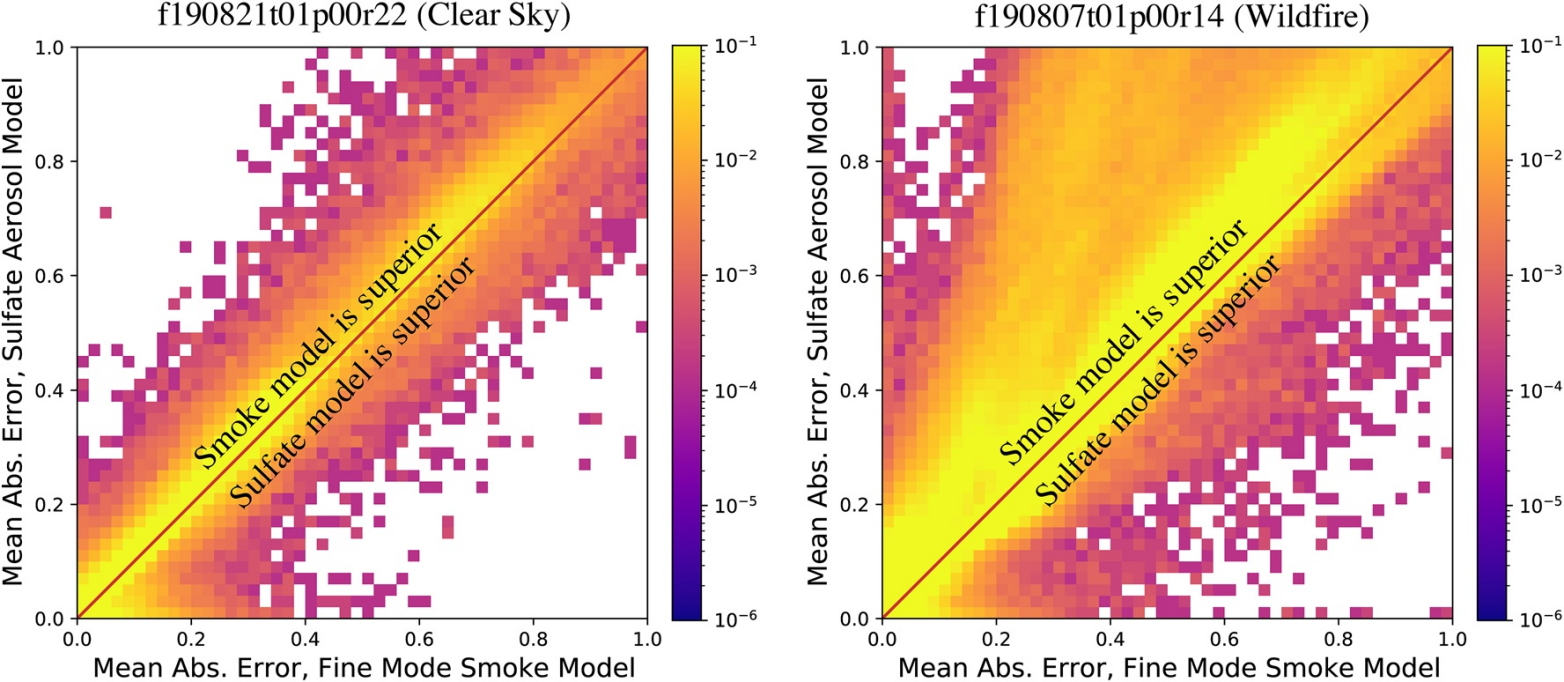
Histogram density of radiance residuals for smoke and sulfate aerosol models, for clear sky and wildfire flightlines.

We further assess the capacity to distinguish between aerosol types by evaluating the residuals between the observed and modeled at‐sensor radiance, using both the smoke and sulfate aerosol models. Figure 7 shows this comparison for two different flight lines (one clear sky, and one wildfire example), using 2D histograms. In the clear sky case (left panel), the majority of points lie on or near the 1:1 line, indicating that both models provide similarly good fits. In a wildfire case (right panel), most points lie well above the 1:1 line, indicating that the smoke model significantly outperforms the sulfate model for these pixels. This provides statistical evidence for the ability of VSWIR imaging spectroscopy from AVIRIS‐C to discriminate aerosol types over heterogeneous scenes.

**Figure 8:**
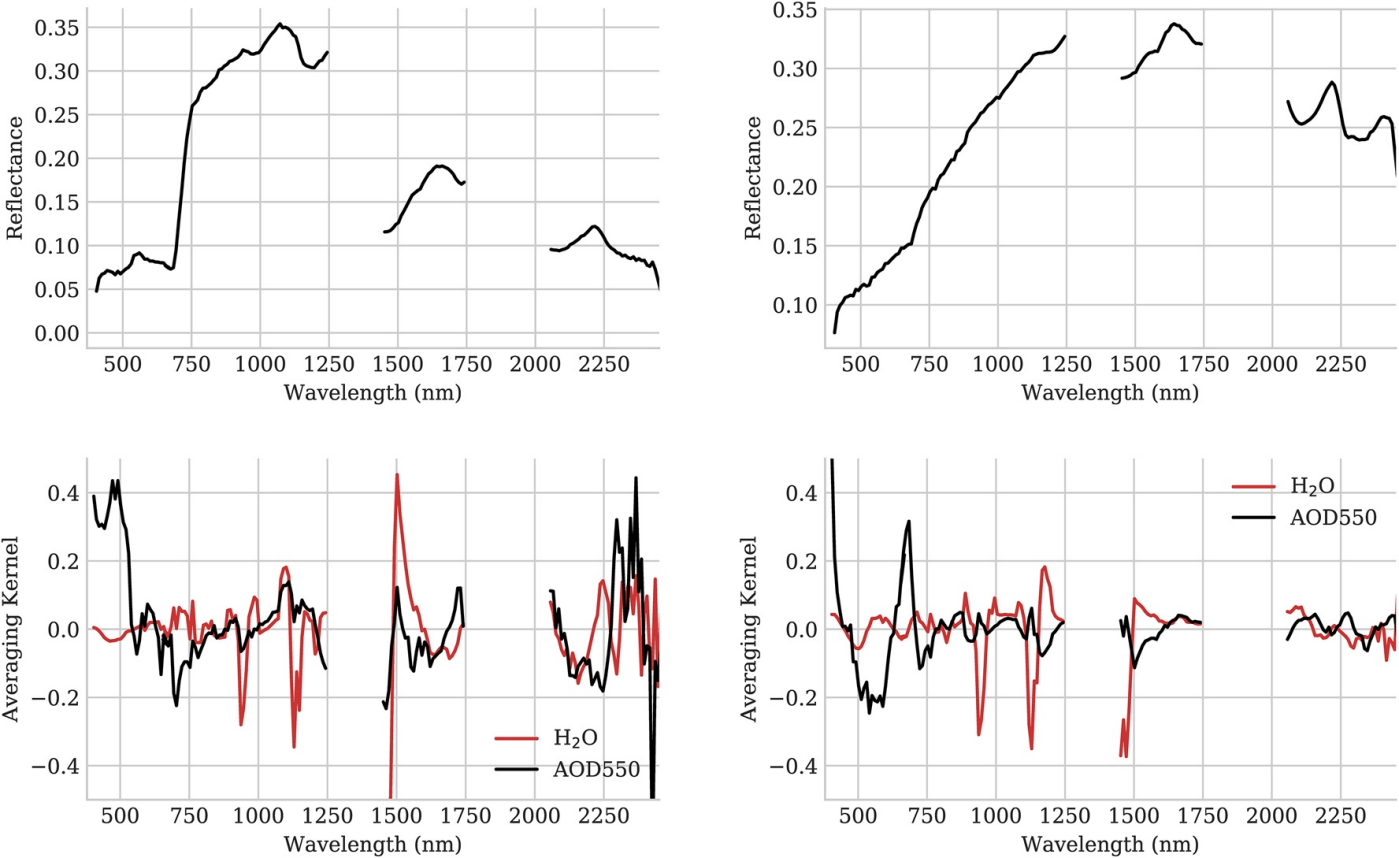
Left: Reflectance spectrum and aerosol averaging kernel for a vegetated pixel from flightline f190807t01p00r14. Right: Reflectance spectrum and aerosol averaging kernel for a bare soil pixel from the same flightline.

Figure 8 shows one example retrieval under thick smoke conditions. The left panel shows the reflectance of a mixed pixel from flightline f190807t01p00r14 along with the averaging kernels corresponding to the H_2_O and AOD550 state variables. The averaging kernel represents the sensitivity of the cost function to the true state by illustrating the impulse response of the final retrieval estimate to a unit perturbation of the rest of the state vector (Rodgers, 2000).

This provides insight into where the inversion draws its information‐values farther from zero (either positive or negative) indicate stronger influence.

The red features, indicating sensitivity to H_2_O, follow the shape of atmospheric absorption features at 940 and 1,140 nm. Interestingly, the edge of the deep absorption feature at 1,480 nm also contributes strongly to the water vapor retrieval. The upslope in the black AOD550 averaging kernel at 500 nm indicates that higher radiances in these channels are interpreted as path radiance, and increase the estimated aerosol. Shortwave channels also contribute to the aerosol estimate, because the surface reflectance of green vegetation is strongly constrained in this region; additional radiance in the low‐signal areas near the opaque water absorption features would be interpreted as an increase in the estimated aerosol load. Lacking a commensurate increase in the contrast of vegetation features in the visible wavelengths, a higher AOD would be required to produce the measured radiance. In contrast, the near infrared portion of the spectrum from 800 to 1,250 nm can vary in brightness due to changes in vegetation reflectance itself, which is more variable in this region. Consequently, the averaging kernel in this area is nearly flat. The right panel of Figure 8 shows a spectrum that contains mostly soil and nonphotosynthetic vegetation. Here, the long wavelengths are unconstrained and contribute little information to either atmospheric parameter. The aerosol retrieval thus relies on the shortest channels; an increase in signal at the shortest wavelengths is attributed to aerosols rather than reflectance.

**Figure 9:**
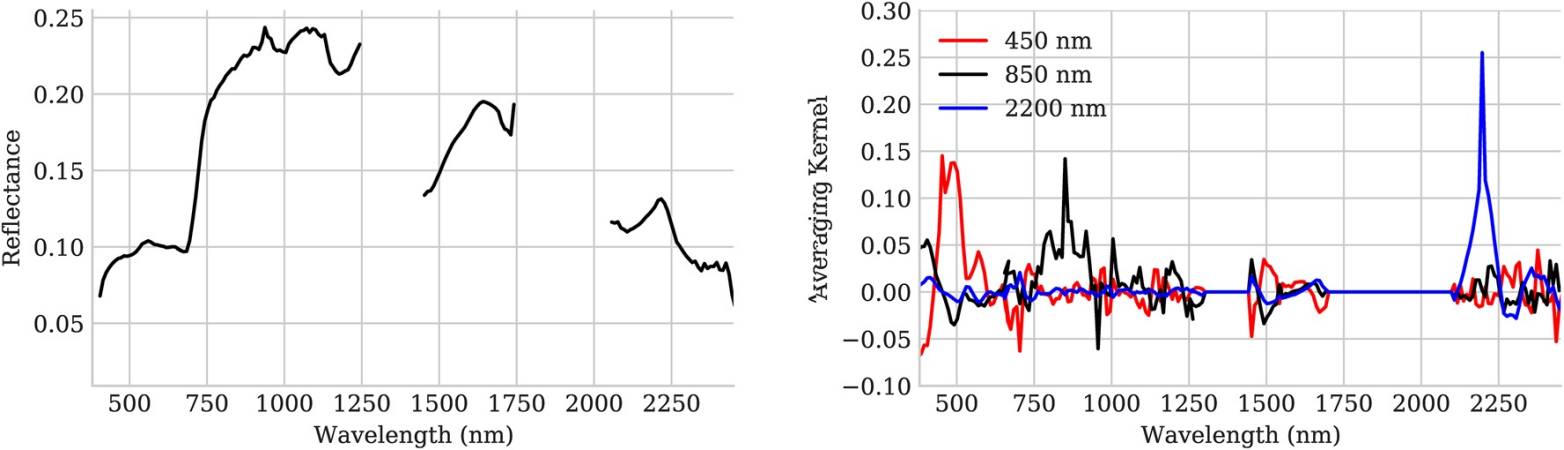
Left: Reflectance spectrum from f190807t01p00r14. Right: The associated averaging kernels for three reflectance channels.

The averaging kernels for individual reflectance channels are also informative. Figure 9 shows those associated with the reflectance retrieval in selected visible, near infrared, and SWIR channels of a smoky scene. The visible wavelength channels are highly influenced by aerosols, reducing the spectral sensitivity of these measurements and broadening the associated averaging kernels. The retrieval of these reflectance values relies on a wide range of wavelengths, leading to nonzero values across the spectrum. In contrast, the SWIR averaging kernel, where the atmosphere is more transparent, is strongly peaked around its associated radiance channel. This reinforces the intuition that, in heavy aerosol loading conditions, the retrieval does not infer the visible wavelength reflectances entirely from the obscured channels, but rather exploits information distributed over the entire the spectrum.

**Figure 10:**
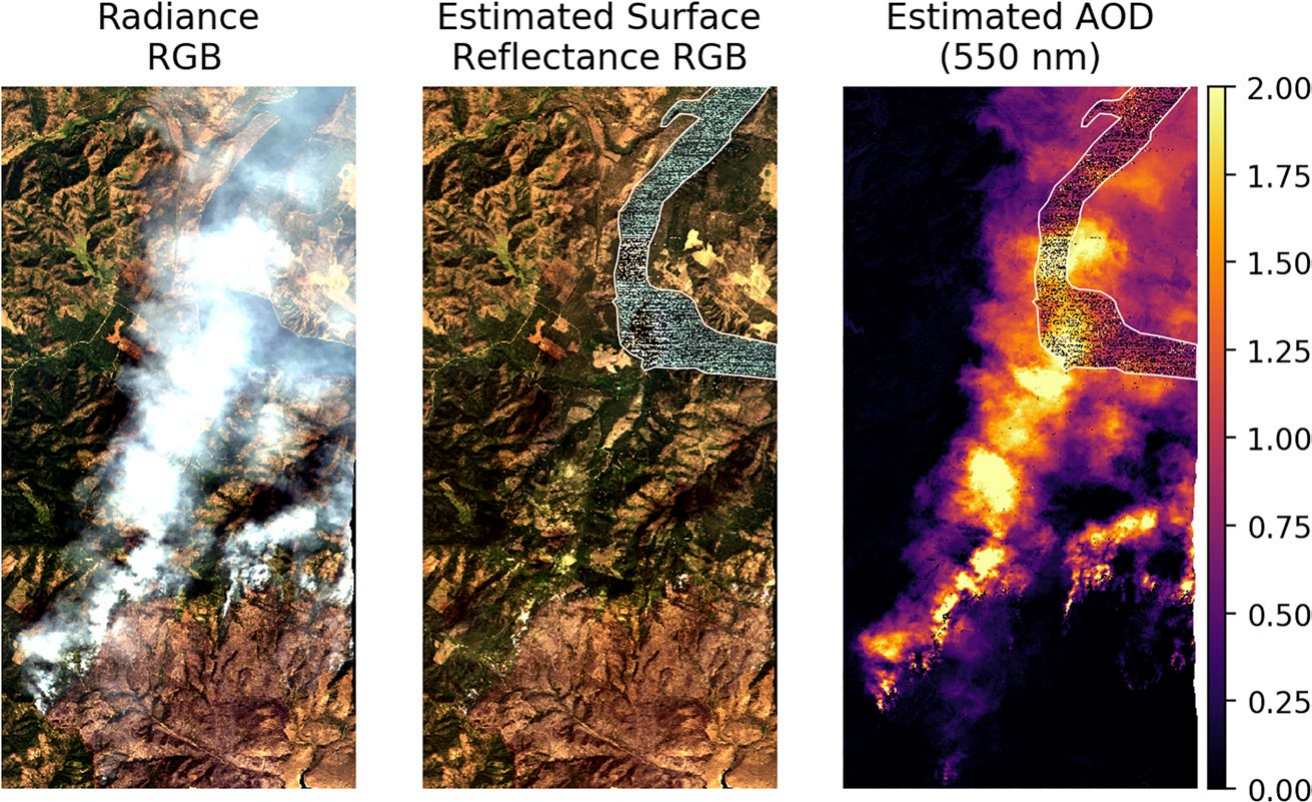
Map of the primary plume near the fire front in flightline f190806t01p00r18. A white line in the upper right denotes a river with a lower surface reflected signal and subsequent relatively poor retrievals.

Finally, we demonstrate how this process can be used to characterize smoke plumes from fires. In Figure 10, we show this retrieval process over an actively burning portion of the Williams Flats Fire near Spokane, WA (Junghenn Noyes et al., 2020). This scene demonstrates how the combination of high spectral fidelity measurements and strong upwind surface priors facilitate retrievals of and through thick smoke, with AODs reaching above 2. Notably, retrievals through smoke over water do not work as well (noticeable in the inconsistent values shown in the river in the upper right corner of the scene). This is due to the weak reflectance of water across the majority of the spectrum, and subsequent low at‐sensor radiance signal, which also amplify any sensor noise effects. However, Figure 10 shows smooth results over a range of surface terrain, with few false positives outside of the plume.

The high spatial resolution mapping of AOD enables a unique characterization of plume dynamics. We fit the second order structure function *S*_2_r, which reveals how the concentration changes as a function of distance from the source. Specifically it describes the expected value of the squared difference in the AOD field *f*(*i*), indexed by location *i*, as a function of separation distance *r* between pairs of points.

(9) $$S_{2}\left(r\right)=E\left[|f\left(i+r\right)-f\left(i\right){|}^{2}\;\right]$$

**Figure 11:**
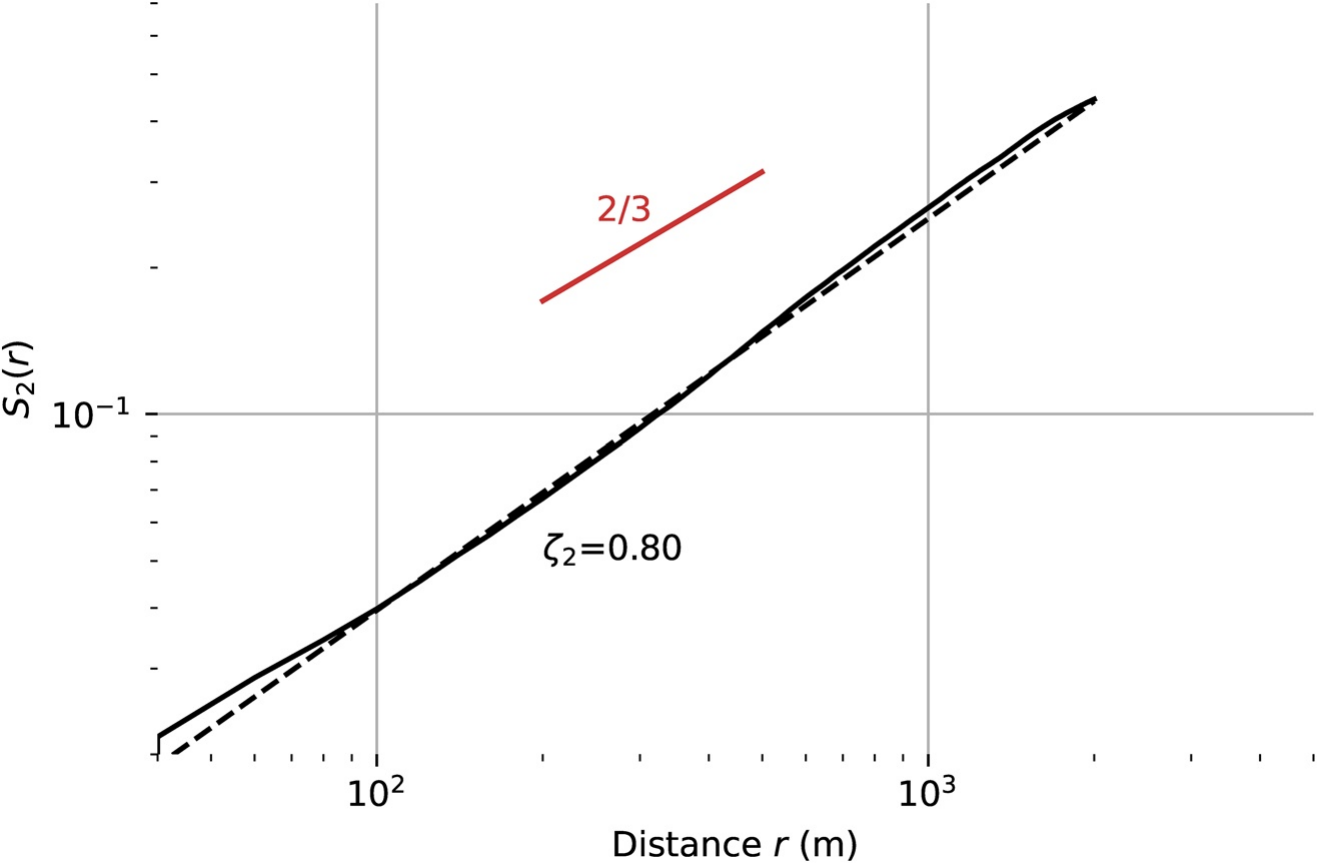
Second order structure function calculated from the particle concentration of the smoke plume in Figure 10. The empirically determined slope of 0.8 is close to the theoretical value of 0.66 that would occur for a passive tracer in turbulent flow.

*S*_*n*_(*r*) is estimated using the mean of observed AOD values at different spatial offsets. It is typically described locally by a power law:

(10) $$S_{2}\left(r\right)\propto r^{\zeta_{2}}$$

where *ζ*_2_ is the second order scaling exponent. Following the Kolmogorov theory, a passive tracer in turbulence has a theoretical second‐order scaling exponent *ζ*_2_ of 2/3 (Pope, 2000). We fit a structure function to image f190806t01p00r18, using an AOD threshold of 0.2 to effectively segment the plume from the background (Figure 11). The second order scaling exponent, identified by the best fitting line in logarithmic space, has a value of 0.8 which is quite close to the theoretical result of 0.66 for a passive turbulent flow. In other words, the small‐scale structure of the plume observed over scales of 50 m to over 1,000 m is broadly consistent with expectation for a turbulent atmosphere. The ideal slope of 2/3 is plotted in red for reference.

## 4 Discussion

Understanding the intensity, distribution, and composition of aerosols is of critical importance to Earth system science and public health. We present a method for using imaging spectroscopy to quantify both aerosol category and optical depth from imaging spectroscopy. Our approach leverages a combined solution of the surface and atmospheric state, facilitating AOD retrievals over dense smoke plumes as well as the characterization of the surface reflectance near active fires‐paving the way for science at the interface of the surface and atmosphere. We demonstrate the efficacy of this method by comparison to ground‐based estimates of AOD, and apply the method to the Williams Flat Fire near Spokane, WA in order to generate a high spatial resolution map of smoke aerosols.

Our procedure uses OE to independently solve for the complete atmospheric and surface state at each pixel, leveraging radiative transfer modeling, calibrated at‐sensor radiance measurements, and an estimate of the surface prior. Due to the reduced surface signal under dense plumes, stronger local priors than commonly utilized (e.g., Carmon et al., 2020; Thompson et al., 2018; Thompson et al., 2020, Thompson, Babu et al., 2019), help inform an accurate retrieval. Deriving these stronger local priors is straightforward, given the increasing quantities of imaging spectroscopy data available. With future orbital imaging spectroscopy missions, generalized sets of strong local priors are likely, particularly given that they may also aid in model uncertainty propagation. Evidence that the algorithm utilizes the full VSWIR spectral range to estimate AOD (Figure 8), including higher wavelengths where aerosols do not have a dominant absorption signature, highlights that these strong priors play a substantial role in the retrieval.

While we were able to demonstrate strong agreement between AOD measured from the ground (AERONET) and remotely (AVIRIS‐C), some discrepancies remained even with our best aerosol model. Several factors could contribute to this. First, while measurements were aligned in time and space to the maximum possible extent, misalignment—particularly in measured optical path—may still be a factor. Additionally, our analyses indicated that accurate AOD retrievals are quite sensitive to absolute radiometric calibration. While we used a vicarious calibration to reduce radiometric calibration errors in AVIRIS‐C data, some calibration errors inevitably remain, and could contribute to observed differences. And finally, and perhaps most significantly, any and all radiative transfer models contain a host of modeling and input data assumptions, and despite our best efforts it is quite possible that these differing assumptions contribute to the observed discrepancies.

Our approach demonstrates the capacity to distinguish between aerosol types, using residuals between modeled and observed radiances. This capacity is critical for global acquisitions, where manual distinctions based on local context will not be feasible due to high data volume rates. Future work will be needed to explore the retrieval capacity of additional aerosol types, within‐class drivers of optical property variation, and aerosol mixtures. Investigations into the influence of different vertical distributions of aerosols, as well as the interaction of aerosols with other trace gases, also remain to be explored.

Smoke has diverse optical properties (Samset et al., 2018). The goal here was simply to show spectroscopic discrimination between broad aerosol categories, which has not to our knowledge been demonstrated for an instrument of this spatial resolution. Nevertheless we recognize the within‐class variance of smoke optical properties—due to the balance of particle sizes and the ratio of black to organic carbon—as a potential source of error in our AOD estimates. We leave the discrimination and measurement of these finer classes to future work.

## 5 Conclusion

With increased global and repeat acquisitions of imaging spectroscopy pending through missions like the EMIT, the SBG mission, and the ACCP, imaging spectroscopy will provide a promising avenue to provide global estimates of aerosol quantity and composition. We do note that our technique performs relatively poorly over aquatic regions, due to strong absorption of light at wavelengths exceeding one micron, but appears to work well over different terrestrial substrates. Future extensions of this work could consider utilizing vertical profile distributions to approximate air quality at the surface, extending the diversity of aerosol types considered, and investigating the relationship between surface characteristics and point source emissions.

## Data Availability

All airborne acquisitions used in this manuscript may be found on the Airborne Visible/Infrared Imaging Spectrometer data portal (https://aviris.jpl.nasa.gov/dataportal/). All retrievals were performed using the open source optimal estimation package ISOFIT v2.9.2 (Thompson et al., 2021).
